# Motor Skill Learning Is Associated with Phase-Dependent Modifications in the Striatal cAMP/PKA/DARPP-32 Signaling Pathway in Rodents

**DOI:** 10.1371/journal.pone.0140974

**Published:** 2015-10-21

**Authors:** Yu Qian, Hans Forssberg, Rochellys Diaz Heijtz

**Affiliations:** 1 Department of Neuroscience, Karolinska Institutet, Retzius Väg 8, SE-171 77, Stockholm, Sweden; 2 Department of Women’s and Children’s Health, Karolinska Institutet, SE-171 76, Stockholm, Sweden; INSERM / CNRS, FRANCE

## Abstract

Abundant evidence points to a key role of dopamine in motor skill learning, although the underlying cellular and molecular mechanisms are still poorly understood. Here, we used a skilled-reaching paradigm to first examine changes in the expression of the plasticity-related gene Arc to map activity in cortico-striatal circuitry during different phases of motor skill learning in young animals. In the early phase, Arc mRNA was significantly induced in the medial prefrontal cortex (mPFC), cingulate cortex, primary motor cortex, and striatum. In the late phase, expression of Arc did not change in most regions, except in the mPFC and dorsal striatum. In the second series of experiments, we studied the learning-induced changes in the phosphorylation state of dopamine and cAMP-regulated phosphoprotein, 32k Da (DARPP-32). Western blot analysis of the phosphorylation state of DARPP-32 and its downstream target cAMP response element-binding protein (CREB) in the striatum revealed that the early, but not late, phase of motor skill learning was associated with increased levels of phospho-Thr34-DARPP-32 and phospho-Ser133-CREB. Finally, we used the DARPP-32 knock-in mice with a point mutation in the Thr34 regulatory site (i.e., protein kinase A site) to test the significance of this pathway in motor skill learning. In accordance with our hypothesis, inhibition of DARPP-32 activity at the Thr34 regulatory site strongly attenuated the motor learning rate and skilled reaching performance of mice. These findings suggest that the cAMP/PKA/DARPP-32 signaling pathway is critically involved in the acquisition of novel motor skills, and also demonstrate a dynamic shift in the contribution of cortico-striatal circuitry during different phases of motor skill learning.

## Introduction

The capacity to learn new motor skills is fundamental for our daily activities and our ability to adapt to challenging environments throughout life. Although learned motor skills can vary widely in type and complexity, all are acquired through repetitive practice, refinement, and storage (for a review, see [1]). The acquisition of new motor skills is characterized by an initial phase of rapid improvement in performance, followed by a later phase of more gradual improvement when performance reaches nearly asymptotic levels at the same time as the skills are consolidated. Once a skill is mastered, it can be readily retrieved with reasonable levels of performance even after extended practice-free periods.

Motor training and motor learning have recently been emphasized as important components of rehabilitation programs for children with motor disabilities ([2] for review). Several studies have demonstrated that children and adolescents with cerebral palsy (CP) can improve their hand functioning by intense training of the impaired hand [3, 4]. There has been increasing interest in understanding the cellular and molecular mechanisms underlying the different phases of motor skill learning, in order to develop new treatment strategies for optimizing motor function in rehabilitation programs.

Various behavioral tasks and experimental paradigms have been used in exploring the neurobiological basis of motor skill learning in rodents, including accelerating rotarod and skilled reaching tasks. These studies have revealed that motor skill learning induces functional changes in the primary motor cortex (M1). In rats, learning a novel skilled reaching task is associated with expanded distal forelimb movement representations in M1 contralateral to the trained forelimb [5]. Evidence also indicates structural changes in white matter microstructure [6], synaptogenesis [7], and spine formation [8]. Studies have shown that the integrity of the mesocortical dopaminergic (DAergic) pathway in M1 is essential for successful motor skill learning and associated synaptic plasticity in this region [9, 10]. A more recent study indicates that dopamine (DA) influences motor skill learning and synaptic plasticity (i.e., long-term synaptic potentiation) in M1 via D1 and D2 receptor–mediated activation of the intracellular phospholipase C (PLC) signaling pathway [11].

A number of human and animal studies indicate that the striatum, the major input nucleus of the basal ganglia, is an important node in the cortico-striatal circuitry subserving motor skill learning [1]. In line with these findings, we found a negative correlation between the motor learning rate and the mRNA levels of dopamine and cAMP-regulated phosphoprotein, 32 kDa (DARPP-32) in both frontal cortex and striatum of mice [12]. DARPP-32 is a signaling hub that plays a critical role in the integration of numerous neurotransmitter and neuromodulator signals arriving at dopaminoceptive neurons (for review, see [13]). In addition, it is highly expressed in medium spiny neurons of the striatum and glutamatergic pyramidal neurons of the frontal cortex (albeit at lower levels) [14–17]. The function of DARPP-32 depends on its relative state of phosphorylation at multiple regulatory sites (e.g., Thr34, Thr75 and Ser97) that are regulated by calcium and cAMP signaling pathways [18, 19]. For example, phosphorylation of DARPP-32 at the threonine 34 (Thr34) regulatory site (e.g., via D1 receptor dependent cAMP/protein kinase A pathway) results in the potent inhibition of a multifunctional serine/Thr protein phosphatase-1 (PP-1) that controls the phosphorylation state of several downstream proteins necessary for synaptic plasticity and learning including the transcription factor cAMP response element-binding protein (CREB). However, still very little is known about the role of DARPP-32 in motor skill learning.

The main goals of this study are three-folds: The first goal was to determine whether the striatum is differentially engaged during different phases of motor skill learning. For this purpose, we studied the induction of the plasticity-related gene activity-regulated cytoskeleton-associated protein (Arc; also known as Arg3.1), an immediate-early gene that has been widely used to map brain activity in the rodent brain, and is also modulated by DA [20]. The second goal was to examine whether different phases of motor skill learning are accompanied by distinct patterns in the phosphorylation state of DARPP-32, and its downstream target CREB in areas marked by Arc. Finally, based on the results of the first two series of studies, we wanted to test whether we could impair motor skill learning by blocking the activity of DARPP-32 at the Thr34 regulatory site (i.e., protein kinase A site) using transgenic mice.

## Materials and Methods

### Animals

#### Rats

All experiments were performed in 5-week-old male outbred Wistar rats (Charles River, Sulzfeld, Germany). The animals arrived in the laboratory three weeks before the experiment and were housed together with their mothers in standard plastic cages (GR 900 IVC; Tecniplast, Buguggiate Va, Italy) under controlled temperature, humidity, and light (12:12 h light–dark cycle, lights on at 0700 h) conditions. Food and water were available *ad libitum*. The animals were weaned from their mothers on postnatal day (PD) 21. One week later, animals were randomly assigned to either a skilled reach training group or an active control group (see below) and housed in pairs. Each set of paired rats was separated by a clear Plexiglas partition (containing small holes, 10 mm in diameter) that divided the home cage in half in order to monitor the daily food intake of each individual rat.

#### Mice

Knock-in mice expressing a mutated form of DARPP-32, in which the phosphorylation site for PKA (Thr-34) is substituted with an alanine (DARPP-32 Thr34A mutant mice; courtesy of Prof. Gilberto Fisone and Prof. Paul Greengard) were generated as described in earlier studies [21], and were backcrossed for at least 10 generations on a C57BL/6 background.

All experiments were conducted according to a protocol approved by the Ethics committee on Animal Research, Stockholm North and in accordance with the European Communities Council Directive of 24 November 1986 (86/609/EEC).

### Skilled reaching procedure for rats

#### Single-pellet reaching apparatus

Single-pellet reaching boxes were made of clear Plexiglas (14 cm wide, 45 cm long, and 35 cm high) as previously described [22]. In the centre of each box’s front wall was an open vertical slot 1.0 cm wide that extended from the floor to a height of 13 cm. Outside the wall, in front of the slot, a shelf (width 4.0 cm and length 14 cm) was mounted 3 cm above the floor. Small indentations to hold food pellets were located 2.0 cm from the inside of the front wall, aligned with the edges of the open slot. This distance prevents the animal from retrieving food pellets using its tongue.

#### Feeding and food restriction

Starting on PD 28 and during skilled reach training, rats in both activate and trained groups were put on a reduced diet until they reached 85–90% of their normal body weight. The growth curves of non-food restricted animals were used as reference. To familiarize the rats with the food pellets used for the skilled reaching task, each rat received 30 of these pellets (45-mg precision-weight, purified rodent tablets; TestDiet, St. Louis, MO, USA) 8 h before the daily Purina Rodent Chow ration given one week before training. Once skilled reach training began, and until the end of the motor training, only rodent chow was provided in the home cage.

#### Pre-training and training sessions

Two days before the skilled reach training, the training group rats were placed into the reaching cage with food pellets on the shelf for 15 min (i.e., in front of the indentation, very close to the open slot). This was done to introduce each animal to the training box and to identify the preferred forelimb for reaching/grasping the pellets through the open slot. By the end of the second day, all animals had demonstrated a consistent preference for one forelimb in their attempts to reach/grasp the pellets (>80% of the time). During the subsequent 3 or 12 days of motor training, each animal in the trained groups underwent a daily 15-min training session consisting of 30 discrete trials. For each trial, one pellet was placed into the indentation located 2.0 cm from the inside of the front wall contralateral to the preferred forelimb. Each food pellet was immediately removed from the shelf when the pellet was displaced too far away from the indentation, to prevent additional reaching attempts after an unsuccessful discrete trial. During inter-trial intervals, rats were trained to leave the open slot at the front of the box and walk to the rear wall of the cage to wait a few seconds before returning to the front of the cage for the subsequent trial. This was accomplished by occasionally placing a food pellet close to the rear wall of the cage. In addition, food pellets were not placed on the shelf on semi-randomly selected trials, in order to teach the animals to reach only when a food pellet was present on the front shelf. To control for the potential effects of increased motor activity, animals in the active control groups were placed into the same training cages and trained to walk between the front and rear walls of the cage for 15 min. Food pellets were individually placed on the front floor of the cage, where animals could eat them with either their tongues or paws. Thus, animals in the active control groups received the same number of pellets and were exposed to the same level of general motor activity as animals in the skilled reach training group; however, they never developed skilled reaching movements.

Performance on the skilled reaching task used here is characterized by an initial phase when skilled reach accuracy improves rapidly over the first five days of training, followed by a later slow phase of learning when performance improvement reaches nearly asymptotic levels [7]. Based on this information, we defined days 3 and 12 as the early and late phases of motor skill learning, respectively. Animals were therefore trained for 3 or 12 consecutive days before being evaluated for molecular and biochemical changes during these two phases.

#### Video recording

Reaching performance was video recorded using a Samsung HMX-H100P high-definition camcorder. Skilled reaches were analyzed and evaluated frame-by-frame using Windows Media Player software.

### Skilled reaching procedure for mice

Mice were trained for 12 days in a single-pellet skilled reaching task, as previously described [12].

### Endpoint analysis of skilled-reaching behavior

Reaching behavior was analyzed by measuring first-attempt success, i.e., the success, in percent, with which an animal obtained a food pellet on first advancing a forelimb toward the food. First-attempt success in percent was calculated as: (number of pellets obtained on first advance/30) × 100. The learning rate was calculated by subtracting the average performance of the first 2 days from the average performance of the last 2 days, and divided by the intertrial intervals, as previously described [12].

### Gene expression studies

Thirty minutes after the last skilled reach training session on either the 3rd training day (early phase, EP) or 12th training day (late phase, LP), trained and active control groups (i.e., EP trained, EP active control, LP trained, and LP active control groups; *n* = 5 per group) were killed by decapitation and their brains rapidly removed, frozen on dry ice, and kept at –80°C until used. Coronal sections (20 μm) of the frontal cortex, striatum and cerebellum were prepared on a cryostat and stored at –80°C until used.

#### In situ hybridization

The Arc and DARPP-32 riboprobes were prepared by amplifying a conserved region of Arc (GenBank: NM_018790, 478–1121 bp) and DARPP-32 (GenBank: AY601872, 760–1283 bp), respectively, from a rat brain cDNA library. The amplified cDNA fragments were subcloned into a pCR1II-TOPO vector (Invitrogen, Lidingö, Sweden) and confirmed by nucleotide sequencing. The DA D1 receptor (Drd1) riboprobe was prepared from a 1.373-kb *Bam*HI fragment of the rat Drd1 cDNA [23] and cloned into the *Eco*RI and Sma1 sites of pGEM-3Z. The DA D2 receptor (Drd2) riboprobe was prepared from a *Bsp*MII-A*fl*II fragment corresponding to nearly the entire third intracellular domain of the rat Drd2, which was subcloned into a pGEM-4Z vector [24]. Linearized plasmids were used to synthesize [^35^S]-UTP-labeled riboprobes. *In vitro* transcription was carried out using the MAXIscript™ SP6/T7 kit (Applied Biosystems, Uppsala, Sweden) and ^35^S-UTP (NEG039H; Perkin Elmer, Upplands Väsby, Sweden) according to the manufacturer’s instructions. The transcripts were purified using NucAway™ spin columns (Applied Biosystems, Uppsala, Sweden). Fixation, pre-hybridization, hybridization, and washes were performed essentially as previously described [25]; hybridization was conducted using 2–5 × 10^6^ cpm/section ^35^S-UTP-labelled RNA probe at 55°C for 14–16 h. Non-specific hybridization was determined by incubating sections with the respective ^35^S-UTP-labelled sense probe under conditions identical to those of the antisense probe. Following RNase A washing, treated slides were hydrated, dried, and exposed to ß-Max autoradiographic film (Kodak BioMax MR film, Sigma-Aldrich, Stockholm, Sweden) for 1–14 days to enable visualization of Arc-, Drd1-, Drd2-, and DARPP-32 hybridization. Calibrated [^14^C]-labeled standards (Amersham Biosciences, Uppsala, Sweden) were incorporated into all cassettes to allow for quantification. Films were developed in D-19 developer for 2 min and in a 1:5 dilution of Amfix fixative for 10 min.

#### Image analysis

**Fig 1:**
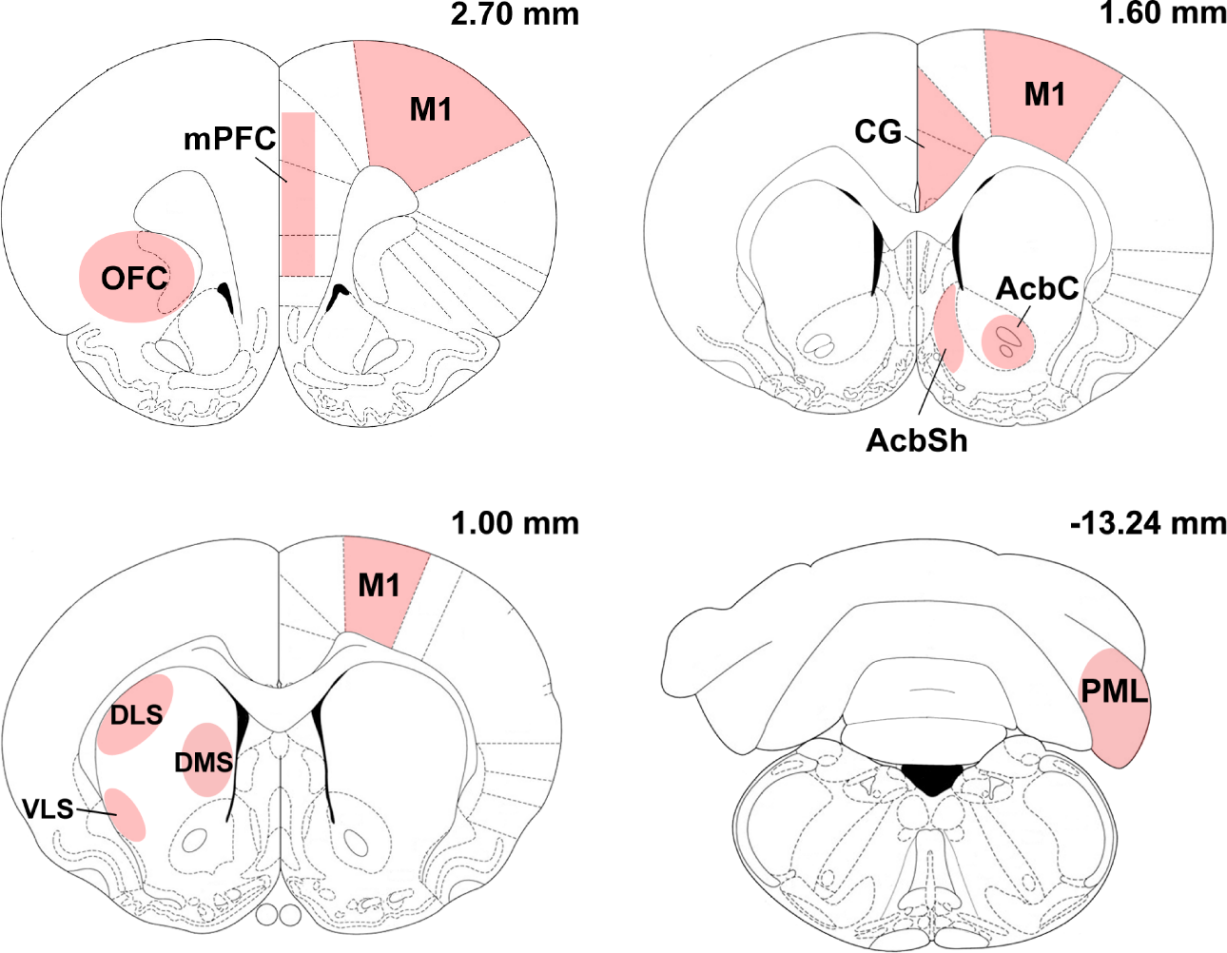
Schematic representation of the brain regions analyzed. Drawings illustrate the location where measures for gene expression studies were obtained. Coronal sections are marked in millimeters from bregma according to the Rat Brain atlas of Paxinos and Watson (1998). Labeled areas correspond to the following brain regions: medial prefrontal cortex (mPFC); orbitofrontal cortex (OFC); cingulate cortex (CG); primary motor cortex (M1); nucleus accumbens core (AcbC); nucleus accumbens shell (AcbSh); dorsal lateral striatum (DLS); dorsal medial striatum (DMS); ventrolateral striatum (VLS); paramedian lobule (PML).

All comparisons between groups were made on sections hybridized together, under identical conditions and exposed for the same time of period to β-Max autoradiographic film. Films were scanned with an Epson Perfection V700 Photo Scanner as grayscale film, using 1200 dpi and saved as high-quality TIFF files. For each animal, the mRNA levels were determined from two to three sections per brain region by measuring the optical density values using the appropriate software (NIH Image J version 1.48, National Institutes of Health). A ^14^C step standard (GE Healthcare, Uppsala, Sweden) was included to calibrate optical density readings and convert measured values into nCi/g. The regions analyzed were the forelimb representation of M1 (2.70 mm to 1.0 mm anterior to bregma), striatum (1.6 mm anterior to -0.26 mm posterior to bregma), nucleus accumbens (1.6 mm to 1.0 mm anterior to bregma), cingulate cortex (1.6 mm to 1.0 mm anterior to bregma), medial prefrontal cortex (2.7 mm to 2.5 mm anterior to bregma), orbital frontal cortex (2.7 mm to 2.5 mm anterior to bregma), and cerebellum (13.68 mm to 12.72 mm posterior to bregma). All brain regions were identified with cresyl-violet-stained sections using an atlas of the rat brain [26] (see Fig 1).

### Biochemical studies

Immediately after the last skilled reach training session on either the 3rd training day (EP group) or 12th training day (LP group), active control and trained groups (i.e., EP trained group, *n* = 8; EP active control group, *n* = 6; LP trained group, *n* = 10; LP active control group, *n* = 6) were killed by decapitation; their brains were rapidly removed and the striata were dissected out within 20 s on an ice-cold surface, frozen immediately on dry ice, and kept at –80°C until used.

#### Western blotting

Samples were homogenized in 700 μL of 1% SDS and 50 mM NaF, and then boiled for 10 min as previously described [14]. Aliquots (10 μL) of the homogenate were used for protein determination using the Quick Start^TM^ Bradford Protein Assay kit (Bio-Rad, Sundbyberg, Sweden). Equal amounts of protein (5–10 μg) for each sample were loaded onto 12% polyacrylamide gels. Proteins were separated by SDS-PAGE and transferred to Immobilon^®^ PVDF membranes (Millipore, Solna, Sweden). The membranes were immunoblotted using polyclonal antibodies against phosho-Thr34-DARPP-32 (cat no.: 12438, 1:1000), phospho-Thr75-DARPP-32 (cat no.: 2301, 1:5000), phospho-Ser-97-DARPP-32 (cat no.: 3401, 1:10,000), and phospho-Ser-133-CREB (cat no.: 9198, 1:10,000; all from Cell Signaling Technology, Beverly, MA, USA). Antibodies against total DARPP-32 and CREB (cat no.: 2306, 1:40,000 and cat no.: 9104, 1:1000, respectively; Cell Signaling Technology, Beverly, MA, USA) that are not phosphorylation state specific were also used to estimate the total amount of these proteins. Antibody binding was revealed by incubation with horseradish peroxidase-conjugated secondary antibodies (Bio-Rad, Sundbyberg, Sweden) and Clarity™ western ECL substrate (Bio-Rad, Sundbyberg, Sweden). Protein band detection was carried out using the ChemiDoc™ XRS+ System with Image Lab™ Software (Bio-Rad, Sundbyberg, Sweden) and quantitated using NIH Image J version 1.29 (National Institutes of Health). Rabbit polyclonal antibody against Heat Shock Protein 90 (cat no.: 4874, 1:10,000; Cell Signaling Technology, Beverly, MA, USA) served as a loading control. At the end of the experiment, nitrocellulose membranes were also stained with Coomassie Blue (Bio-Rad, Sundbyberg, Sweden) to verify the equal loading of proteins.

### Statistical analysis

Statistical analyses were performed using STATVIEW software (version 5.1). All behavioral experiments were analyzed using either repeated measures analysis of variance (ANOVA) or factorial ANOVA when appropriate. Statistical analysis of gene expression studies was performed using one-way ANOVA, considering hemisphere as a covariate). All *post hoc* comparisons were made using a Bonferroni/Dunn test when significant ANOVA effects were found. Phosphorylation data were analyzed with two-tailed unpaired Student's *t*-test. The threshold for statistical significance was set as *P* ≤ 0.05. All data are presented as the mean (M) ± standard error of the mean (SEM).

## Results

### Behavioral assessment of rats used for molecular and biochemical studies

**Fig 2:**
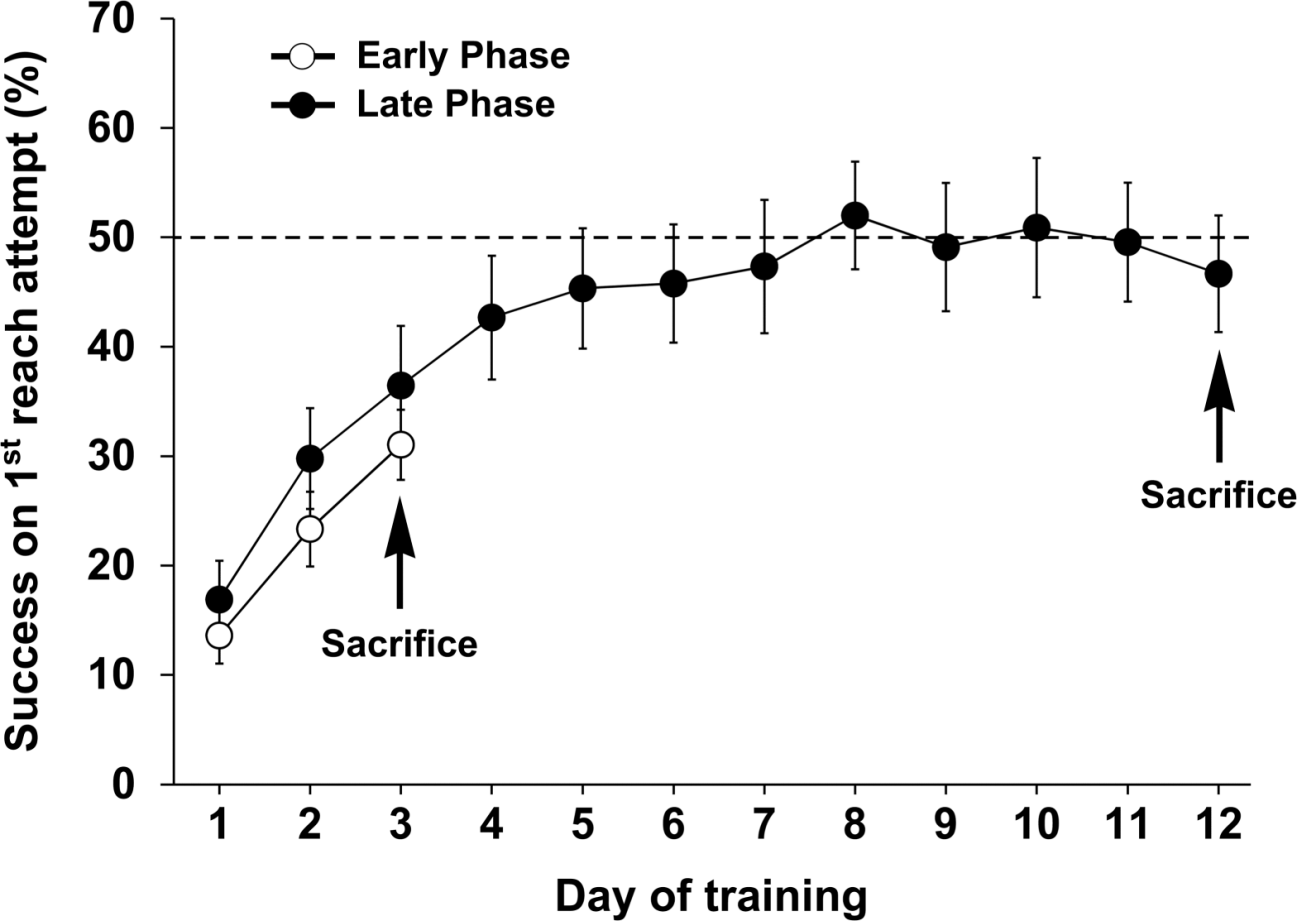
The performance of Wistar rats in the single pellet skilled reaching task used in the present study. The success on the first reach in percentage is presented. To investigate the dynamics of gene expression and biochemical changes at different phases of motor learning, one group of rats, the Early Phase (EP), were trained in the skilled reaching task for 3 days. A second group, the Late Phase (LP), was trained for 12 days (i.e., until asymptotic levels of reaching performance were achieved). The results are presented as the means ± SEM (n = 13–15 per group).

The performance of animals in the skilled reaching task is illustrated in Fig 2. The line graphs represent success on the first reach attempt (i.e., the most sensitive endpoint measure of skilled performance) in percent for the EP (white circles) and LP (black) groups. Repeated-measures ANOVA indicated significant effects of day of training on final reaching accuracy for both the EP (F_(12,24)_ = 17.411; *P* < 0.0001) and LP (F_(14,154)_ = 17.411; *P* < 0.0001) groups. *Post hoc* Bonferroni/Dunn analyses indicated a significant increase in reaching success after 3 days and 12 days of training (*P* < 0.0001) compared with the first day of training. In the EP group, performance improved from 14% (± 3%) on day 1 to 31.0% (± 3%) on day 3. In the LP group, performance improved from 17% (± 3%) on day 1 to 47.0% (± 5%) on day 12. Additional analyses revealed no significant improvements in performance in the LP group over training days 5–12 (*P* > 0.1), indicating that the performance of the LP group (i.e., 12 days of training) had stabilized by the time of killing. There were no significant differences in motor learning between the EP and LP on days 1–3 (F _(1,26)_ = 1.02; *P* = 0.32).

### Patterns of Arc mRNA induction during different phases of motor skill learning

**Fig 3:**
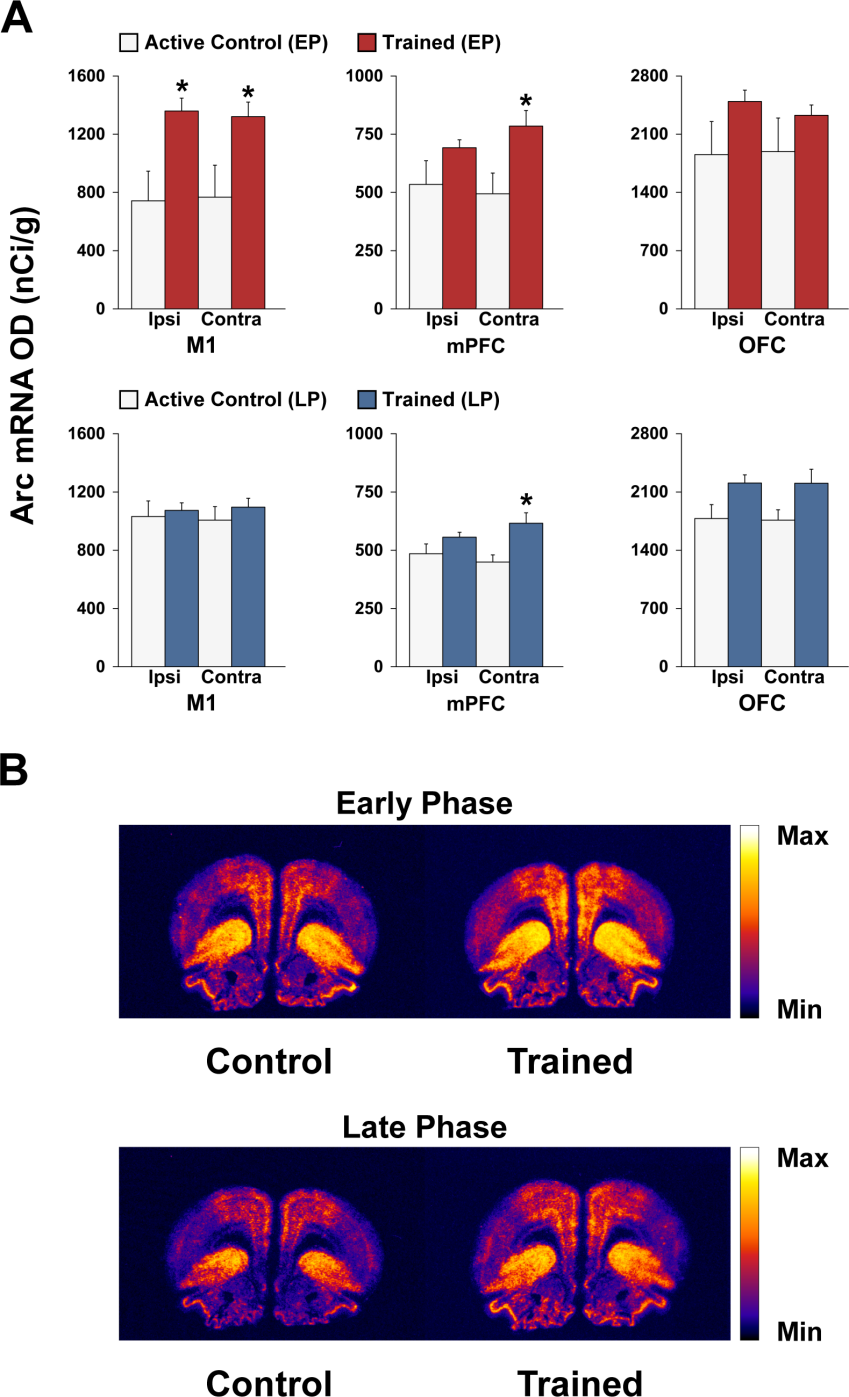
Arc mRNA expression is differentially induced in cortical regions during different phases of motor skill learning. (A) Regional analysis of Arc mRNA expression in various cortical regions of (top panel) early phase (EP) active controls (white bars) vs. EP trained rats (red bars), and of (bottom panel) late phase (LP) active controls (white bars) vs. LP trained rats (blue bars). Data in bar graphs show mean optical density (OD) values for Arc mRNA in M1, mPFC, and OFC. The results are presented as means ± SEM; *n* = 4–5 per group. Asterisks indicate where trained rats differ significantly (*P < 0.05) from active control rats. Abbreviations are as follows: ipsilateral (Ipsi) and contralateral (Contra). (B) Representative autoradiographs showing the mRNA expression levels of Arc at the level of the frontal cortex of active controls and trained rats during the early and late phases of motor skill learning. The pseudo-coloring indicates signal intensity ranging from low (black/purple) to high (yellow/white). For more details, see Figs 1 and 2.

**Fig 4:**
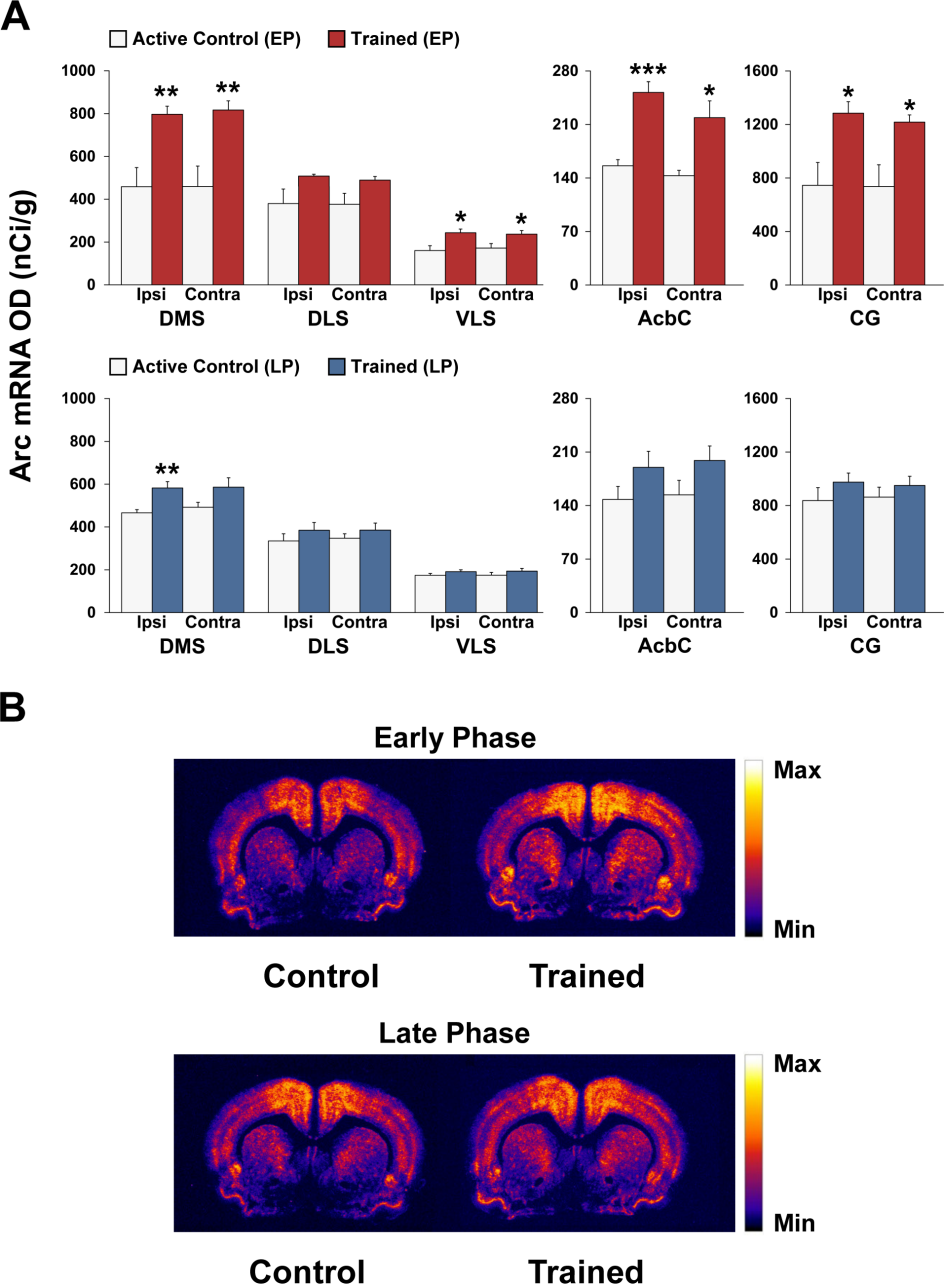
Arc mRNA expression is differentially induced within sub-regions of the striatum during different phases of motor skill learning. (A) Regional analysis of Arc mRNA expression within sub-regions of the striatum of (top panel) early phase (EP) active controls (white bars) vs. EP trained rats (red bars), and of (bottom panel) late phase (LP) active controls (white bars) vs. LP trained rats (blue bars). Data in bar graphs show mean optical density (OD) values for Arc mRNA in DMS, DLS, VLS, AcbC, and AcbSh. The results are presented as means ± SEM; *n* = 5 per group. Asterisks indicate where trained rats differ significantly (*P < 0.05, **P < 0.01, and ***P < 0.001) from active control rats. (B) Representative autoradiographs showing the mRNA expression levels of Arc at the level of the striatum of active controls and trained rats during the early and late phases of motor skill learning. For more details, see Figs 1 and 2.

Figs 3 and 4 show the effects of 3 and 12 days of motor training on Arc mRNA expression in the primary motor cortex (M1), medial prefrontal cortex (mPFC), orbital frontal cortex (OFC), cingulate cortex (CG), striatum, shell (AchSh) and core (AchC) regions of the nucleus accumbens (for anatomical guidance, see Fig 1). Arc expression was highest in the OFC and lowest in the nucleus accumbens (note the different scales of the *y*-axes).

#### Cortical regions

ANOVA for mPFC revealed significant effects of condition (trained vs. active control) for both the EP (F_(1,16)_ = 8.4; *P* < 0.05) and LP (F_(1,16)_ = 10.96; *P* < 0.01) groups. *Post hoc* Bonferroni/Dunn analyses confirmed an increase in Arc gene expression in the mPFC contralateral to the trained forelimb in both the EP and LP trained groups compared with their respective active control groups (*P* < 0.05; Fig 3A), but not in the ipsilateral cortex (*P* > 0.1). For M1 and CG, there were also significant effects of condition [(F_(1,14)_ = 14.79; *P* < 0.01) and (F_(1,14)_ = 18.53; *P* < 0.001), respectively] in the EP groups, but not in the LP groups (*P* > 0.1). *Post hoc* Bonferroni/Dunn analyses indicated an increase in Arc gene expression in both hemispheres of the EP trained group compared with their respective EP active control groups (*P* < 0.05, Figs 3A and 4A, respectively).

#### Striatum

ANOVA for the DMS and VLS revealed significant effects of condition [(F_(1,16)_ = 23.62; *P* < 0.001) and (F_(1,16)_ = 14.41; *P* < 0.01), respectively] in the EP groups. *Post hoc* Bonferroni/Dunn analyses indicated a significant increase in Arc expression in the bilateral DMS and VLS (albeit less robust than in the DMS) of the EP trained group compared with the EP active control group (*P* < 0.01 and *P* < 0.05, respectively; Fig 4A). In contrast, no significant changes were found in the DLS. In the LP trained group, ANOVA revealed a significant effect of condition only in the DMS (F_(1,16)_ = 12.41; *P* < 0.01). Bonferroni/Dunn analyses indicated a small, but significant increase in Arc expression in the ipsilateral DMS of the LP trained group (*P* < 0.01; Fig 4A).

#### Nucleus accumbens

ANOVA for the core region revealed significant effects of condition (F_(1,14)_ = 26.59; *P* < 0.001) in the EP group, but not in the LP trained group (*P >* 0.1). *Post hoc* Bonferroni/Dunn analyses indicated a significant increase in Arc gene expression in the bilateral core region of the EP trained group compared with the EP active control group (*P* < 0.001 and *P* < 0.05, respectively; Fig 4A). No significant effects were found in the shell region in either the EP or LP trained groups (data not shown).

#### Cerebellum

ANOVA for the PML, a region of the cerebellar cortex involved in forelimb movement [27], failed to detect any significant effects (data not shown).

### Dynamic shifts in striatal expression of dopamine-related genes during early and late phases of motor skill learning

**Fig 5:**
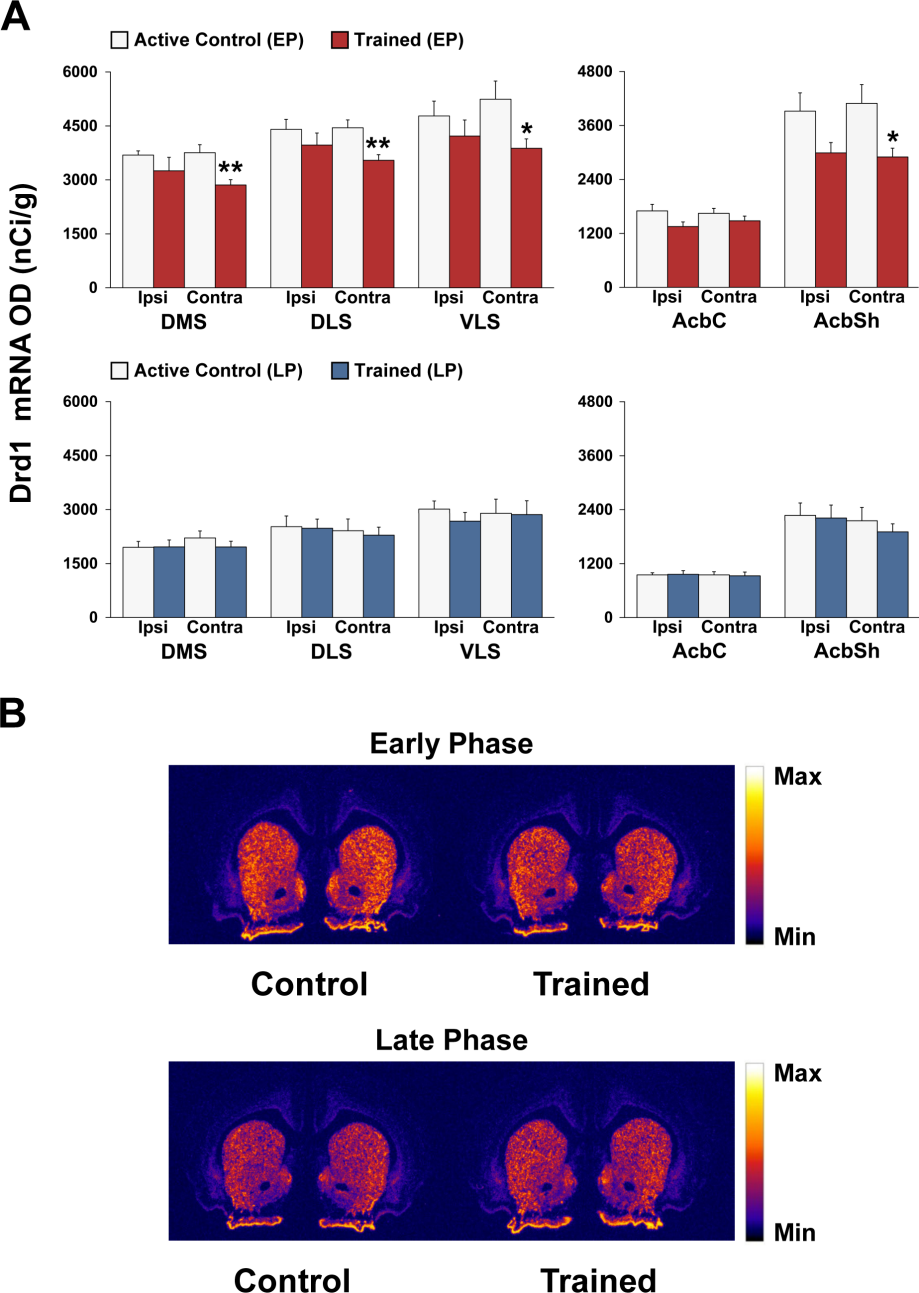
Decreased striatal Drd1 mRNA expression during the early, but not late, phase of motor skill learning. (A) Regional analysis of dopamine D1 receptor (Drd1) mRNA expression within sub-regions of the striatum of (top panel) early phase (EP) active controls (white bars) vs. EP trained rats (red bars), and of (bottom panel) late phase (LP) active controls (white bars) vs. LP trained rats (blue bars). Data in bar graphs show mean optical density (OD) values for Drd1 mRNA in the DMS, DLS, VLS, AcbC, and AcbSh. The results are presented as means ± SEM; *n* = 5 per group. Asterisks indicate where trained rats differ significantly (*P < 0.05 and **P < 0.01) from active control rats. (B) Representative autoradiographs showing the mRNA expression levels of Drd1 at the level of the striatum of active controls and trained rats during the early and late phases of motor skill learning. For more details, see Figs 1 and 2.

**Fig 6:**
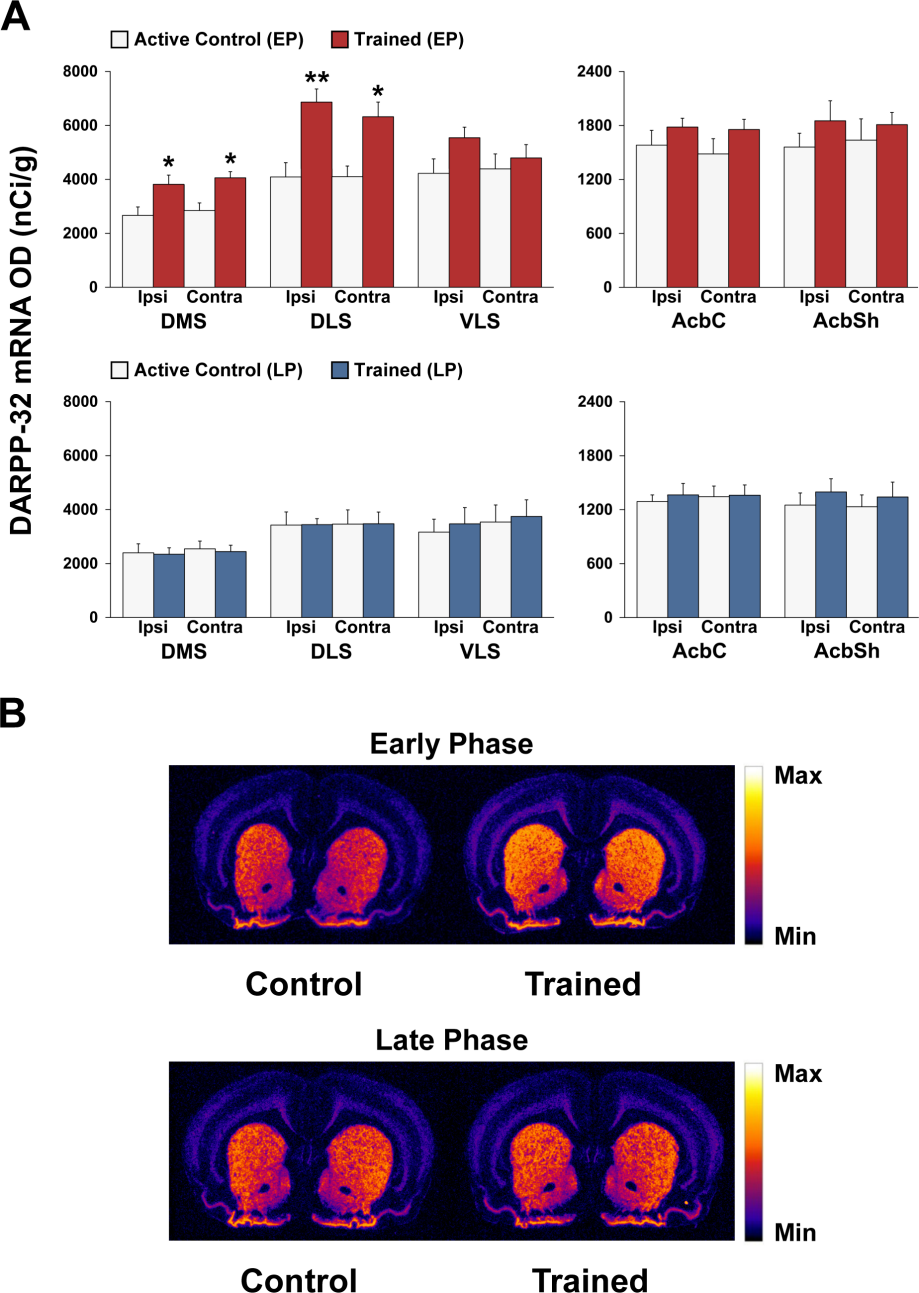
Increased striatal DARPP-32 mRNA expression during the early, but not late, phase of motor skilled learning. (A) Regional analysis of dopamine- and cAMP-regulated neuronal phosphoprotein (DARPP-32) mRNA expression within sub-regions of the striatum of (top panel) early phase (EP) active controls (white bars) vs. EP trained rats (red bars), and of (bottom panel) late phase (LP) active controls (white bars) vs. LP trained rats (blue bars). Data in bar graphs show mean optical (OD) density values for DARPP-32 mRNA in the DMS, DLS, VLS, AcbC, and AcbSh. The results are presented as means ± SEM; *n* = 5 per group. Asterisks indicate where trained rats differ significantly (*P < 0.05 and **P < 0.01) from active control rats. (B) Representative autoradiographs showing the mRNA expression levels of Drd1 at the level of the striatum of active controls and trained rats during the early and late phases of motor skill learning. For more details, see Figs 1 and 2.

Figs 5 and 6 show the effects of 3 or 12 days of motor skill training on Drd1 and DARPP-32 mRNA expression, respectively, in the striatum and nucleus accumbens.

#### Drd1

ANOVA for the DMS, DLS, VLS, and AcbSh revealed significant effects of condition [(F_(1,16)_ = 8.04; *P* < 0.05), (F_(1,16)_ = 6.94; *P* < 0.05), (F_(1,16)_ = 5.38; *P* <0.05) and (F_(1,16)_ = 10.54; *P* < 0.01), respectively] in the EP group, but not in the LP group (*P* > 0.1). *Post hoc* Bonferroni/Dunn analyses indicated a significant decrease in Drd1 gene expression in the DMS, DLS, VLS, and AcbSh contralateral to the trained forelimb of the trained group compared with the active control group (*P* < 0.01, *P* < 0.01, *P* < 0.05 and *P* < 0.05, respectively; Fig 5A). No significant differences were detected in the AchC, mPFC, M1, OFC and CG regions in either the EP or LP trained groups (S1 Table).

#### DARPP-32

ANOVA for the DMS, and DLS revealed significant effects of condition [(F_(1,16)_ = 16.21; *P* < 0.01) and (F_(1,16)_ = 25.74; *P* < 0.001), respectively] in the EP group. *Post hoc* Bonferroni/Dunn analyses revealed a significant increase in DARPP-32 mRNA expression in the bilateral DMS and DLS of the EP trained group compared with the EP active control group (*P* < 0.05; Fig 6A). However, no significant changes were found in the DMS and DLS in the LP trained groups (data not shown). In addition, there were no significant changes in the AchSh and AchC regions (see Fig 6A) or cortical regions (see S1 Table).

#### Drd2

There were no significant changes in Drd2 gene expression after either 3 or 12 days of motor training (*P* > 0.05) (see S2 Table).

### Enhanced phosphorylation of DARPP-32 at the Thr34 site and its downstream target CREB is associated with the early, but not late, phase of motor skill learning

**Fig 7:**
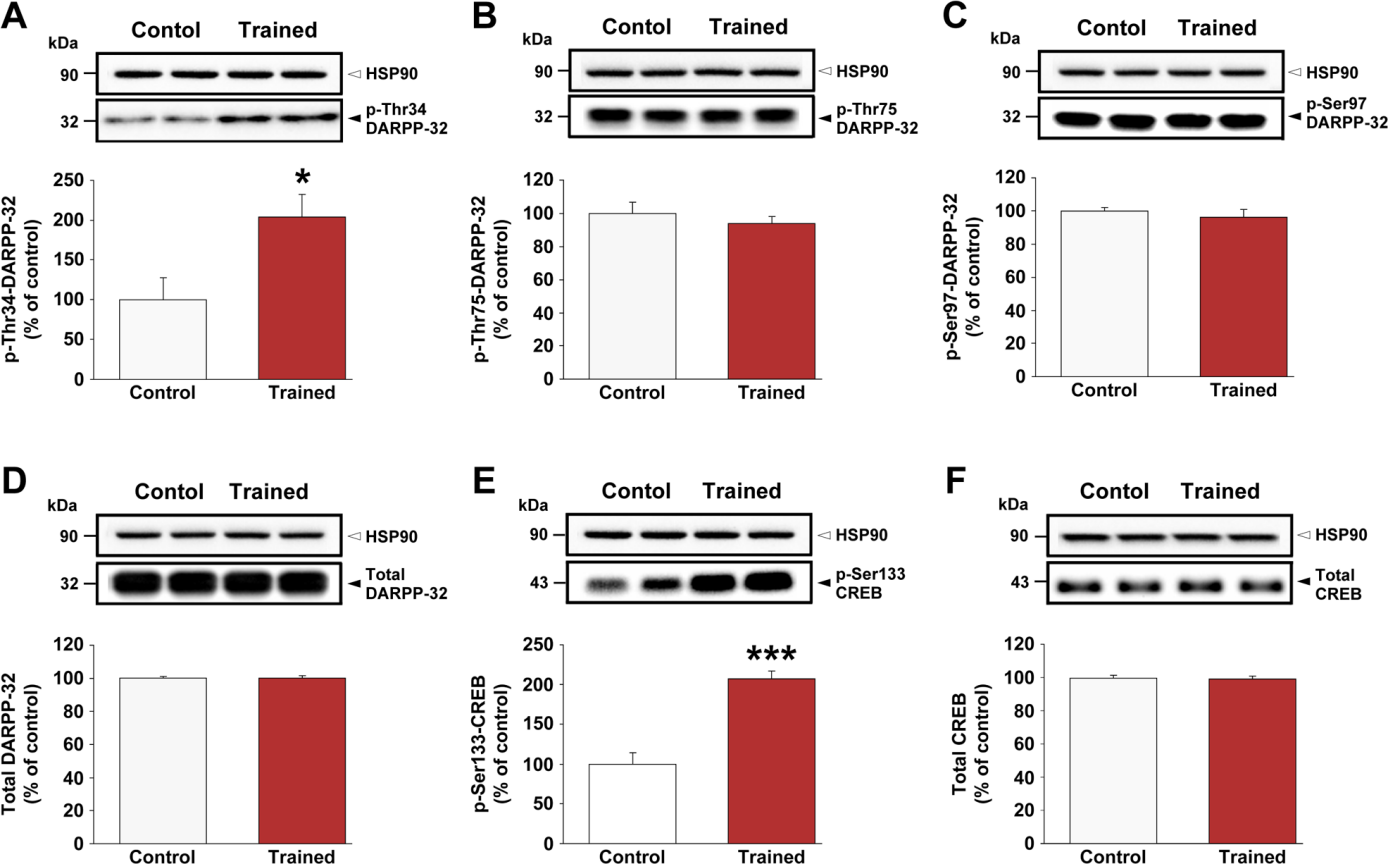
Enhanced phosphorylation of DARPP-32 and CREB in the striatum is associated with the early phase of motor skill learning. (A) Levels of phospho-Thr34-DARPP-32, (B) phospho-Thr75-DARPP-32, (C) phospho-Ser97-DARPP-32, (D) total DARPP-32, (E) phospho-Ser133-CREB, and (F) total CREB were evaluated in the striatum contralateral to the trained forelimb of active controls (white bars) and trained (red bars) rats after 3 days of motor skill learning (i.e., early phase) by means of Western blotting. The top row presents representative autoradiograms. The bottom graph is a summary of data represented as means ± SEM (*n* = 6–8 per group). Asterisks indicate where trained rats differ significantly (*P < 0.05) from active control rats. Heat Shock Protein 90 (HSP90) served as the loading control in A–F.

Examination of total protein levels in the striatum contralateral to the trained forelimb did not reveal differences in DARPP-32 or CREB between the EP trained group and the EP active control group (see Fig 7D and 7F, respectively). In contrast, phospho-Thr34-DARPP-32 and phospho-Ser133-CREB levels were significantly higher (*P* < 0.05 and *P* < 0.001, respectively) in the striatum contralateral to the trained forelimb of the EP trained group compared with the EP active control (see Fig 7A and 7E). The levels of phospho-Thr34-DARPP-32 and phospho-Ser133-CREB, however, did not differ significantly between the LP trained and LP active control groups (data not shown). Moreover, no significant differences were found in the levels of phospho-Thr75-DARPP-32 or phospho-Ser97-DARPP-32 (see Fig 7B and 7C) in either the EP or LP (see S3 Table) trained groups when compared with their respective active control groups.

### Correlations between skilled-reaching performance during the late phase of motor skill learning and levels of phospho-Thr75-DARPP-32 and phospho-Ser133-CREB

**Fig 8:**
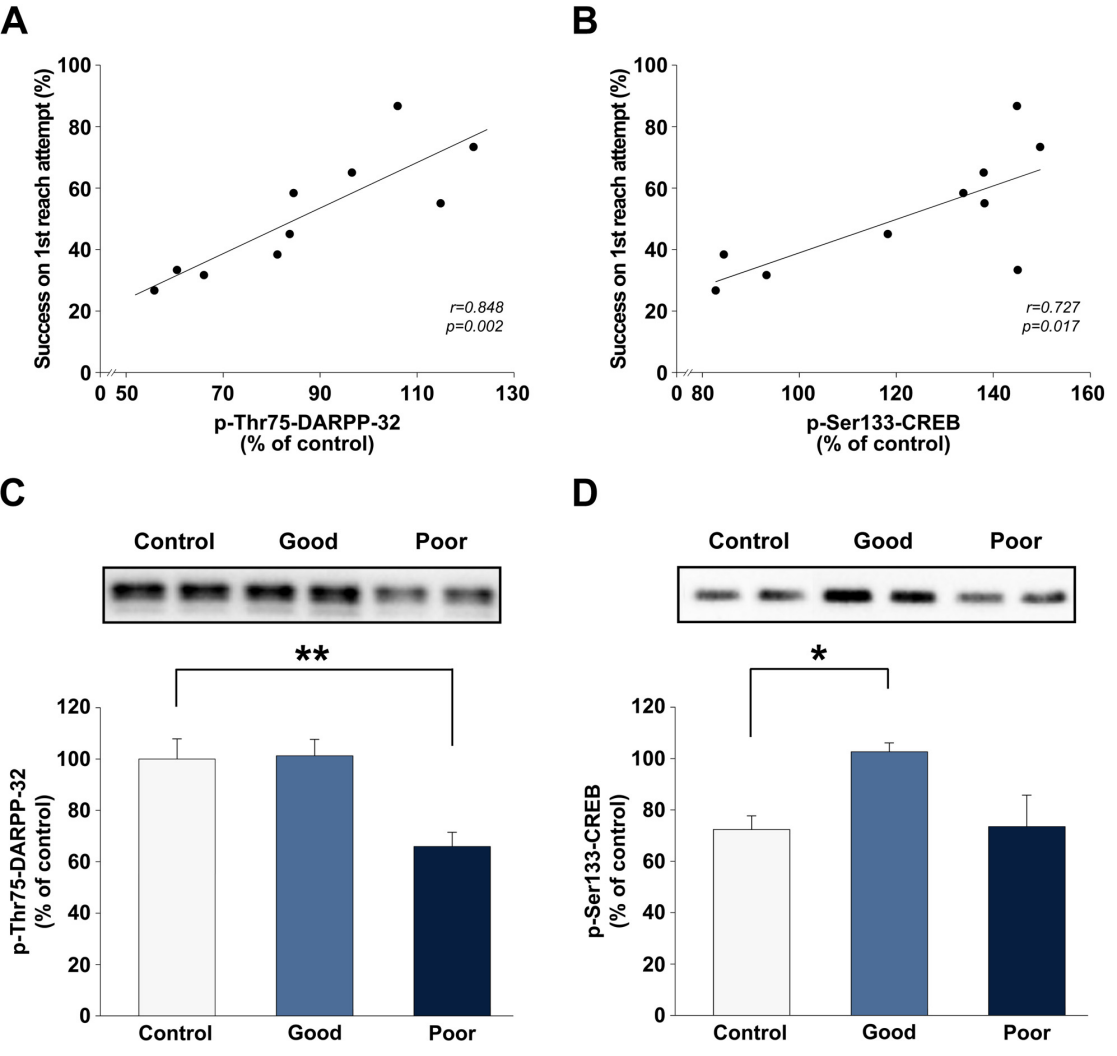
Correlations between skilled-reaching performance during the late phase of motor skill learning and levels of either phospho-Thr75-DARPP-32 or phospho-Ser133-CREB. (A) Simple regression analysis indicating a significant correlation between success on the first reach attempt, in percent, during the late phase of motor skill learning and levels of phospho-Thr75-DARPP-32 or (B) phospho-Ser133-CREB. Filled circles represent values for individual rats from the late phase (LP) group. LP trained rats were divided into good and poor learners according to their individual learning curve profiles and endpoint performance. (C-D) The top row presents representative Western blot autoradiograms for phospho-Thr75-DARPP-32 and phospho-Ser133-CREB. (C) Data in bar graphs represent the levels of phospho-Thr75-DARPP-32 or (D) phospho-Ser133-CREB in controls (*n* = 6), good learners (*n* = 6), and poor learners (*n* = 4) expressed as means ± SEM. The asterisks indicate where poor or good learners differ significantly (*P < 0.05) from active controls.

In a recent study, we found a negative correlation between the rate of motor learning and level of DARPP-32 expression in the bilateral striata [12]. We therefore examined the possibility that variation in skilled-reaching performance during the late phase of motor skill learning would be associated with levels of phospho-DARPP-32 at specific regulatory sites, i.e., Thr34, Thr75, and Ser97. A highly significant positive correlation was found between skilled-reaching performance (i.e., average success on the first reach attempt on days 11 and 12) and level of phospho-DARPP-32 at the Thr75 site, but not at the Thr34 or Ser97 sites (*r* = 0.848, *P* = 0.002; Fig 8A), in the striatum contralateral to the trained forelimb. In a subsequent analysis, rats were divided into either good or poor learners according to their individual learning curve profiles and performance during the last 5 days of training. A good learner was defined as an animal that had a clear learning curve (i.e., displaying an incremental level of performance over training days 1–5) and an average or above level of performance during the last 5 days of training (i.e., days 8–12). A poor learner was defined as an animal that did not display a clear learning curve and had a level of performance 50% lower than the average. Analysis of the state of DARPP-32 phosphorylation according to levels of performance (i.e., good vs. poor) indicated that the levels of phospho-Thr75-DARPP-32 were significantly lower in the poor learner group compared with the active control group (*P* < 0.01; Fig 8C).

Given that CREB is a downstream target of DARPP-32 that has also been implicated in the molecular cascade that mediates the formation of long-lasting memories, we explored a potential correlation between skilled-reaching performance and levels of CREB at the Ser133 site. A highly significant positive correlation was found between skilled-reaching performance and level of phospho-Ser133-CREB (*r* = 0.727, *P* = 0.017; Fig 8B). Analysis of the state of CREB phosphorylation according to level of performance (i.e., good vs. poor) revealed that the levels of phospho-Ser133-CREB were significantly higher in the good learner group compared with the active control group (*P* < 0.05; Fig 8D).

### Impaired motor skill learning in knock-in DARPP-32 mice with a mutated Thr34 regulatory site

**Fig 9:**
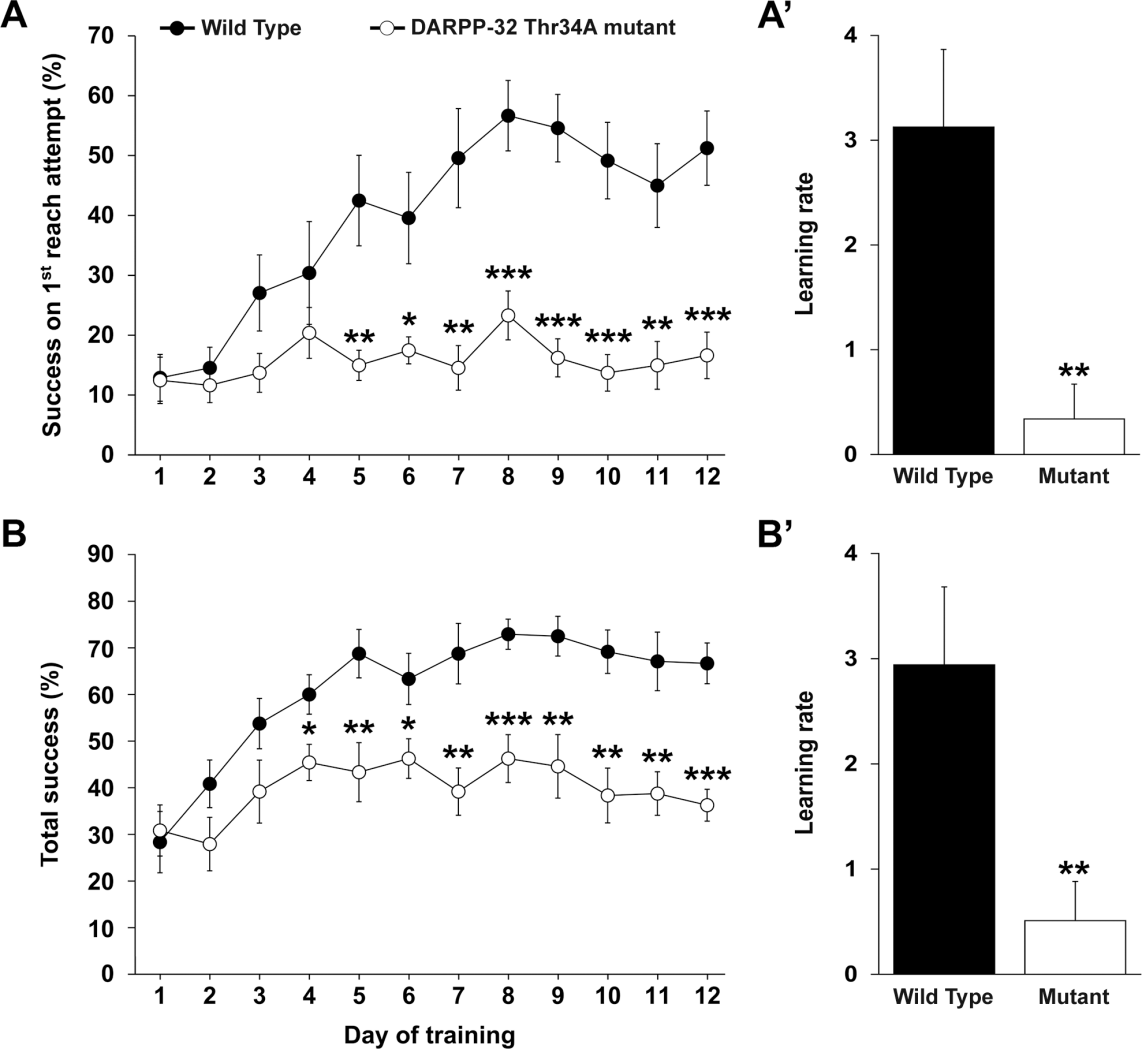
Deficits in skilled reaching behavior in DARPP-32 Thr34A mutant mice. Endpoint measurements of reaching behavior are presented. (A) Success on the first reach attempt, in percentage. (B) Total success, in percentage. (A′ and B′) Bar graphs show the learning rate for each endpoint. The results are presented as means± SEM; n = 8 per group. Asterisks denote where DARPP-32 Thr34A mutant mice differ significantly (* *P* < 0.05, ** *P* < 0.01, *** *P* < 0.001) from wild type (WT) mice.

Young adult DARPP-32 Thr-34 mutant mice and wild type (WT) mice were trained for 12 days in a single pellet skilled reaching as previously described [12]. Fig 9 illustrates the skilled reaching behavior of adult T34A DARPP-32 mutant mice and wild type mice (WT) mice. Repeated-measures anova for success on the first reach attempt (%) showed significant effects of strain (*F*_1,14_ = 21.05, *P* < 0.001), training day (*F*_11,154_ = 9.08, *P* < 0.0001) and a strain by training day interaction (*F*_11,154_ = 5.83, *P* < 0.0001). The DARPP-32 Thr-34 mutant mice displayed little improvement over the 12-day testing period (Fig 9A). *Post hoc* Bonferroni/Dunn analyses confirmed that DARPP-32 Thr-34 mutant mice had significantly (*P* < 0.05) lower scores in success on the first reach attempt (%) on days 5–12 of training compared with wild type (WT) mice. Moreover, DARPP-32 Thr-34 mutant mice had a significantly (*P* < 0.01) lower learning rate than WT mice (Fig 9A’).

Analysis of total success (%) by repeated-measures anova showed significant effects of strain (*F*_1,14_ = 17.65, *P* < 0.001), training day (*F*_11,154_ = 10.39, *P* < 0.0001) and a strain by training day interaction (*F*_11,154_ = 3.10, *P* < 0.001). *Post hoc* Bonferroni/Dunn analyses showed that DARPP-32 Thr-34 mutant mice had significantly lower scores in total success on days 4–12 compared with WT mice (*P* < 0.05; Fig 9B), as well as lower learning rate (*P* < 0.01; Fig 9B’).

## Discussion

This study demonstrates that training in a novel skilled reaching task during early acquisition engages a cortico-striatal circuitry, which becomes more restricted as the skill is automatized. Moreover, the early, but not late, phase of motor skill learning is associated with alterations in the expression of DA-related genes and with changes in intracellular signaling pathways. Furthermore, inhibition of DARPP-32 activity at the PKA site (Thr34) impaired skilled motor learning. Our studies suggest that activation of the cAMP/PKA/DARPP-32 pathway in the striatum is critically involved in fine motor skill acquisition.

The immediate early gene Arc has been widely implicated in synaptic and experience-dependent plasticity. A unique characteristic of Arc is that its mRNA traffics to dendrites and accumulates at activated synapses [28]. Although the function of Arc is still unclear, it is believed to be part of a versatile, finely-tuned system capable of coupling changes in neuronal activity to diverse forms of synaptic plasticity, such as long-term potentiation (LTP), long-term depression (LTD), and homeostatic plasticity, thereby optimizing information storage in active networks [28]. In the present study, we demonstrate that Arc mRNA is induced within a bilateral cortico-striatal network of young animals, including M1, mPFC (roughly homologous to the dorsolateral PFC in humans), CG, and the striatum during the early phase of motor learning. These findings are consistent with human neuroimaging studies demonstrating recruitment of similar cortical regions (e.g., M1 and dorsolateral PFC) during the initial stages of motor skill learning (for a review, see [1]). Involvement of M1 during the early phase of motor skill learning has been well documented in adult rodents. For example, recruitment of neurons in M1 occurs in behaving mice during the initial phases of learning the rotarod task [29]. More recently, imaging studies in adult mice also demonstrated that training in a skilled reaching task leads to rapid formation (within an hour) of dendritic spines on pyramidal neurons in M1 contralateral to the trained forelimb [8]. In the present study, we found that Arc mRNA was induced in both the contralateral and ipsilateral M1 during the early phase of motor skill learning. The changes observed in ipsilateral M1 are not entirely unexpected given that rodents coordinate their non-preferred forelimb to support the body during reaching. Moreover, evidence from human studies indicates that the primary motor cortex is activated bilaterally during skilled unimanual movements [30].

We also found that Arc mRNA was induced in various dorsal striatum sub-regions, particularly in the DMS or associative striatum, which receives input primarily from association cortices such as the prefrontal cortex. These findings are consistent with studies in rats demonstrating that inhibiting protein synthesis in the dorsal striatum impairs the acquisition of skilled reaching [31], and with electrophysiological studies in monkeys and rodents suggesting a more prominent role for the associative striatum during the early phase of motor skill learning [32, 33]. This is also in agreement with functional neuroimaging studies in humans and electrophysiological experiments in monkeys and rodents have implicated the striatum in motor skill learning (for a review, see [1]).

Arc mRNA was also induced in the ventral striatum (i.e., core and shell regions of the nucleus accumbens) during the early phase of learning. Although there is limited direct evidence for a role of the nucleus accumbens in motor skill learning, it is tempting to speculate a role in the motivational processes affecting motor learning (for a review, see [34]). Interestingly, training-induced changes in Arc mRNA expression were not detectable in the PML, a cerebellar cortex region involved in forelimb movement, suggesting less involvement of cortico-cerebellar circuits for acquisition of skilled forelimb movements [27]. The cortico-cerebellar circuits, however, may be more important in other types of motor skill learning e.g., involving the coordination of all four limbs [27, 35].

A striking finding of this study is the dramatic shift observed in the Arc expression pattern in the cortico-striatal circuits during the various phases of motor skill learning, with substantially lower expression during the late phase. These observations are consistent with electrophysiological studies in mice indicating differential cortico-striatal plasticity (e.g., neuronal firing rate) during the early and late phases of motor skill learning [29].

DA is a neurotransmitter that plays a major role in motor learning and synaptic plasticity. For example, learning of a rotarod task is impaired by moderate disruption of DA release in the striatum by the neurotoxin 6-OHDA [36] and by downregulation of synaptotagmin I (a protein involved in neurotransmitter release) in the nigrostriatal terminals [37]. In addition, it has been demonstrated that destruction of DAergic terminals from the ventral tegmental area to cortex or blockade of DA receptors in M1 impairs motor skill acquisition, but not the execution of a previously acquired motor skill (for a review, see [38]). In humans, studies using molecular positron emission tomography (PET) imaging have found changes in striatal dopamine release during the initial acquisition of a motor skill [39]. Moreover, indications suggest that genetic variation in the human DA system influences motor skill learning and modulatory responses to L-DOPA [40].

In the present study, we examined training-induced plasticity changes of the dopamine system at multiple levels (from gene expression to biochemical changes in intracellular signaling pathways). At the mRNA level, our data demonstrate training-induced changes in the expression of DA D1 receptors and their intracellular target DARPP-32 in the striatum during the early, but not late, phase of motor skill learning. The results of western blot analysis, however, failed to reveal any changes in total DARPP-32 and DA D1R (data not shown) protein levels. One reason might be that the animals used for the biochemical studies were sacrificed immediately after the task (i.e., an optimal time point for detecting changes in protein phosphorylation), while later time points would have been more optimal for changes in total protein levels. No alterations in DA D2 receptor expression were found, unlike reports regarding the striatum of rats after circling training (i.e., gross motor training) [41]. Our results are consistent with our previous human PET studies demonstrating that the training of working memory is associated with changes in the density of cortical DA D1 but not D2 receptors [42].

In the present study, we further investigated the potential involvement of DARPP-32, a signaling “hub” that integrates information arriving at dopaminoceptive neurons via a variety of neurotransmitters and neuromodulators [43]. In particular, DARPP-32 is crucial for the expression of striatal synaptic plasticity, for example, LTP and LTD [44]. Our biochemical studies revealed that the early, but not late, phase of motor skill learning is associated with increased phosphorylation of DARPP-32 in the striatum, specifically at the Thr34 site. Moreover, we demonstrated that the acquisition and performance of skilled reaching behavior were strongly attenuated in DARPP-32 knock-in mice with a point mutation in the Thr34 regulatory site (i.e., protein kinase A site). Previous studies have demonstrated that activation of DA D1 receptors results in protein kinase A (PKA)-catalyzed phosphorylation of DARPP-32 at the Thr34 site [45]. Together with previous studies demonstrating that mice lacking LTP specifically in the striatum are impaired in motor skill learning [46], the present results suggest that the striatal DA D1 receptor/cAMP/PKA/DARPP-32 pathway is important for acquisition of new fine motor skills.

At the cellular level, when DARPP-32 is phosphorylated at the Thr34 site it becomes a potent inhibitor of PP-1, which controls the phosphorylation state and hence the physiological activity of several downstream targets including CREB [47]. CREB family transcription factors have been implicated in hippocampal-dependent learning and memory (for a review, see [48]) and in cortico-striatal synaptic plasticity [49]. Moreover, mice with a mutation in the CREB-binding (KIX) domain of the CREB-binding protein exhibit impaired motor learning in a rotarod paradigm [50]. Consistent with these findings, we also found an increase in phosphorylation of CREB on Ser-133 during the early, but not late, phase of motor skill learning. In addition, we found a positive correlation between individual skilled-reaching performance during the late phase of motor learning and levels of phospho-Ser133-CREB, with a significant increase in phosphorylation in the striatum of good learners relative to controls. This suggests that an optimal level of sustained CREB phosphorylation is required during the late phase of motor skill learning for successful consolidation of motor memories. The absence of any correlations with the levels of PKA-mediated phosphorylation of DARPP-32 at the Thr34 site indicates that other signaling pathways (e.g., Ca^2+^/calmodulin-dependent kinase IV and mitogen-activated protein kinases) may be modulating the state of CREB phosphorylation during the late phase of motor skill learning [51]. Interestingly, we also found a strong positive correlation between skilled reaching performance and levels of phospho-Thr75-DARPP-32, poor learners having lower levels of phosphorylation relative to controls. It has previously been demonstrated that cyclin-dependent kinase 5 (Cdk5) phosphorylates DARPP-32 at the Thr75 site, converting DARPP-32 into a PKA inhibitor [52]. Under basal conditions, the levels of phospho-Thr75-DARPP-32 in the striatum are high and DAergic signaling is constrained by tonic activity of Cdk5 signaling. On the other hand, DA acting through the D1 receptor/cAMP/PKA pathway decreases the state of phosphorylation of striatal DARPP-32 at the Thr75 site, thereby removing the Cdk5-mediated inhibitory constraint [53]. We postulate that there might be an imbalance in other neurotransmitters and second messengers involved in phosphorylating Cdk5 or its regulatory factors in poor learners, since we failed to observe enhanced PKA-mediated phosphorylation of DARPP-32 at the Thr34 site during the late phase of motor skill learning.

In a recent study, Rioult-Pedotti and collaborators reported that DAergic signaling in M1 influences motor skill learning and synaptic-plasticity via activation of the intracellular PLC pathway, but not the PKA pathway [11]. In contrast, the results of the present study provide compelling evidence for the involvement of the striatal cAMP/PKA/DARPP-32 signaling pathway in the acquisition of novel motor skills. The reasons for the discrepancies between our studies are unclear, but they could be related to regional differences in the expression of DA receptor subtypes. For example, DA D5 receptors, which are coupled to the PLC signaling pathway, are more abundantly expressed in cortical regions than in the striatum [54–57]. An important challenge for future studies is to unravel the specific role of DA receptor subtypes within cortico-striatal circuitry in motor skill learning, and to identify the key downstream targets required for successful motor skill learning.

## Supporting Information

S1 Table Expression of Drd1 and DARPP-32 mRNAs in various cortical regions after 3 or 12 days of motor skill learning. (PDF)

S2 Table Expression of Drd2 mRNA in various brain regions after 3 or 12 days of motor skill learning. (PDF)

S3 Table Levels of total and phosphorylated DARPP-32 and CREB in striatum after 12 days of motor skill learning. (PDF)

## References

[1] Dayan E, Cohen LG. Neuroplasticity subserving motor skill learning. Neuron. 2011;72(3):443–54. 10.1016/j.neuron.2011.10.008 22078504 PMC3217208

[2] Novak I, McIntyre S, Morgan C, Campbell L, Dark L, Morton N, et al A systematic review of interventions for children with cerebral palsy: state of the evidence. Developmental medicine and child neurology. 2013;55(10):885–910. 10.1111/dmcn.12246 . 23962350

[3] Bonnier B, Eliasson AC, Krumlinde-Sundholm L. Effects of constraint-induced movement therapy in adolescents with hemiplegic cerebral palsy: a day camp model. Scand J Occup Ther. 2006;13(1):13–22. . 16615411 10.1080/11038120510031833

[4] Gordon AM, Hung YC, Brandao M, Ferre CL, Kuo HC, Friel K, et al Bimanual training and constraint-induced movement therapy in children with hemiplegic cerebral palsy: a randomized trial. Neurorehabil Neural Repair. 2011;25(8):692–702. 10.1177/1545968311402508 . 21700924

[5] Kleim JA, Barbay S, Nudo RJ. Functional reorganization of the rat motor cortex following motor skill learning. Journal of neurophysiology. 1998;80(6):3321–5. . 9862925 10.1152/jn.1998.80.6.3321

[6] Sampaio-Baptista C, Khrapitchev AA, Foxley S, Schlagheck T, Scholz J, Jbabdi S, et al Motor skill learning induces changes in white matter microstructure and myelination. The Journal of neuroscience: the official journal of the Society for Neuroscience. 2013;33(50):19499–503. 10.1523/JNEUROSCI.3048-13.2013 24336716 PMC3858622

[7] Kleim JA, Hogg TM, VandenBerg PM, Cooper NR, Bruneau R, Remple M. Cortical synaptogenesis and motor map reorganization occur during late, but not early, phase of motor skill learning. The Journal of neuroscience: the official journal of the Society for Neuroscience. 2004;24(3):628–33. 10.1523/JNEUROSCI.3440-03.2004 . 14736848 PMC6729261

[8] Xu T, Yu X, Perlik AJ, Tobin WF, Zweig JA, Tennant K, et al Rapid formation and selective stabilization of synapses for enduring motor memories. Nature. 2009;462(7275):915–9. 10.1038/nature08389 19946267 PMC2844762

[9] Molina-Luna K, Pekanovic A, Rohrich S, Hertler B, Schubring-Giese M, Rioult-Pedotti MS, et al Dopamine in motor cortex is necessary for skill learning and synaptic plasticity. PloS one. 2009;4(9):e7082 10.1371/journal.pone.0007082 19759902 PMC2738964

[10] Hosp JA, Pekanovic A, Rioult-Pedotti MS, Luft AR. Dopaminergic projections from midbrain to primary motor cortex mediate motor skill learning. The Journal of neuroscience: the official journal of the Society for Neuroscience. 2011;31(7):2481–7. 10.1523/JNEUROSCI.5411-10.2011 . 21325515 PMC6623715

[11] Rioult-Pedotti MS, Pekanovic A, Atiemo CO, Marshall J, Luft AR. Dopamine Promotes Motor Cortex Plasticity and Motor Skill Learning via PLC Activation. PloS one. 2015;10(5):e0124986 10.1371/journal.pone.0124986 25938462 PMC4418826

[12] Qian Y, Chen M, Forssberg H, Diaz Heijtz R. Genetic variation in dopamine-related gene expression influences motor skill learning in mice. Genes, brain, and behavior. 2013;12(6):604–14. 10.1111/gbb.12062 . 23819855

[13] Svenningsson P, Nishi A, Fisone G, Girault JA, Nairn AC, Greengard P. DARPP-32: an integrator of neurotransmission. Annual review of pharmacology and toxicology. 2004;44:269–96. 10.1146/annurev.pharmtox.44.101802.121415 . 14744247

[14] Kuroiwa M, Snyder GL, Shuto T, Fukuda A, Yanagawa Y, Benavides DR, et al Phosphodiesterase 4 inhibition enhances the dopamine D1 receptor/PKA/DARPP-32 signaling cascade in frontal cortex. Psychopharmacology. 2012;219(4):1065–79. 10.1007/s00213-011-2436-8 21833500 PMC3539205

[15] Perez RG, Lewis RM. Regional distribution of DARPP-32 (dopamine- and adenosine 3',5'-monophosphate-regulated phosphoprotein of Mr = 32,000) mRNA in mouse brain. The Journal of comparative neurology. 1992;318(3):304–15. 10.1002/cne.903180307 . 1533862

[16] Ouimet CC, LaMantia AS, Goldman-Rakic P, Rakic P, Greengard P. Immunocytochemical localization of DARPP-32, a dopamine and cyclic-AMP-regulated phosphoprotein, in the primate brain. The Journal of comparative neurology. 1992;323(2):209–18. 10.1002/cne.903230206 . 1328330

[17] Ouimet CC, Miller PE, Hemmings HC Jr., Walaas SI, Greengard P. DARPP-32, a dopamine- and adenosine 3':5'-monophosphate-regulated phosphoprotein enriched in dopamine-innervated brain regions. III. Immunocytochemical localization. The Journal of neuroscience: the official journal of the Society for Neuroscience. 1984;4(1):111–24. . 6319625 10.1523/JNEUROSCI.04-01-00111.1984 PMC6564746

[18] Girault JA. Integrating neurotransmission in striatal medium spiny neurons. Advances in experimental medicine and biology. 2012;970:407–29. 10.1007/978-3-7091-0932-8_18 . 22351066

[19] Walaas SI, Hemmings HC Jr., Greengard P, Nairn AC. Beyond the dopamine receptor: regulation and roles of serine/threonine protein phosphatases. Frontiers in neuroanatomy. 2011;5:50 10.3389/fnana.2011.00050 21904525 PMC3162284

[20] Gangarossa G, Di Benedetto M, O'Sullivan GJ, Dunleavy M, Alcacer C, Bonito-Oliva A, et al Convulsant doses of a dopamine D1 receptor agonist result in Erk-dependent increases in Zif268 and Arc/Arg3.1 expression in mouse dentate gyrus. PloS one. 2011;6(5):e19415 10.1371/journal.pone.0019415 21559295 PMC3086923

[21] Svenningsson P, Tzavara ET, Carruthers R, Rachleff I, Wattler S, Nehls M, et al Diverse psychotomimetics act through a common signaling pathway. Science. 2003;302(5649):1412–5. 10.1126/science.1089681 . 14631045

[22] Vergara-Aragon P, Gonzalez CL, Whishaw IQ. A novel skilled-reaching impairment in paw supination on the "good" side of the hemi-Parkinson rat improved with rehabilitation. The Journal of neuroscience: the official journal of the Society for Neuroscience. 2003;23(2):579–86. . 12533618 10.1523/JNEUROSCI.23-02-00579.2003 PMC6741884

[23] Schambra UB, Duncan GE, Breese GR, Fornaretto MG, Caron MG, Fremeau RT Jr. Ontogeny of D1A and D2 dopamine receptor subtypes in rat brain using in situ hybridization and receptor binding. Neuroscience. 1994;62(1):65–85. . 7816213 10.1016/0306-4522(94)90315-8

[24] Bunzow JR, Van Tol HH, Grandy DK, Albert P, Salon J, Christie M, et al Cloning and expression of a rat D2 dopamine receptor cDNA. Nature. 1988;336(6201):783–7. 10.1038/336783a0 . 2974511

[25] Diaz Heijtz R, Fuchs E, Feldon J, Pryce CR, Forssberg H. Effects of antenatal dexamethasone treatment on glucocorticoid receptor and calcyon gene expression in the prefrontal cortex of neonatal and adult common marmoset monkeys. Behavioral and brain functions: BBF. 2010;6:18 10.1186/1744-9081-6-18 20307270 PMC2858712

[26] Paxinos G, Watson C. The Rat Brain: In Stereotaxic Coordinates: Academic Press, Incorporated; 1998.

[27] Santori EM, Der T, Collins RC. Functional metabolic mapping during forelimb movement in rat. II. Stimulation of forelimb muscles. The Journal of neuroscience: the official journal of the Society for Neuroscience. 1986;6(2):463–74. . 3950706 10.1523/JNEUROSCI.06-02-00463.1986 PMC6568535

[28] Bramham CR, Alme MN, Bittins M, Kuipers SD, Nair RR, Pai B, et al The Arc of synaptic memory. Experimental brain research. 2010;200(2):125–40. 10.1007/s00221-009-1959-2 19690847 PMC2803749

[29] Costa RM, Cohen D, Nicolelis MA. Differential corticostriatal plasticity during fast and slow motor skill learning in mice. Current biology: CB. 2004;14(13):1124–34. 10.1016/j.cub.2004.06.053 . 15242609

[30] Ehrsson HH, Fagergren A, Jonsson T, Westling G, Johansson RS, Forssberg H. Cortical activity in precision- versus power-grip tasks: an fMRI study. Journal of neurophysiology. 2000;83(1):528–36. . 10634893 10.1152/jn.2000.83.1.528

[31] Wachter T, Rohrich S, Frank A, Molina-Luna K, Pekanovic A, Hertler B, et al Motor skill learning depends on protein synthesis in the dorsal striatum after training. Experimental brain research. 2010;200(3–4):319–23. 10.1007/s00221-009-2027-7 . 19823812

[32] Miyachi S, Hikosaka O, Lu X. Differential activation of monkey striatal neurons in the early and late stages of procedural learning. Experimental brain research. 2002;146(1):122–6. 10.1007/s00221-002-1213-7 . 12192586

[33] Yin HH, Mulcare SP, Hilario MR, Clouse E, Holloway T, Davis MI, et al Dynamic reorganization of striatal circuits during the acquisition and consolidation of a skill. Nature neuroscience. 2009;12(3):333–41. 10.1038/nn.2261 19198605 PMC2774785

[34] Ena S, de Kerchove d'Exaerde A, Schiffmann SN. Unraveling the differential functions and regulation of striatal neuron sub-populations in motor control, reward, and motivational processes. Frontiers in behavioral neuroscience. 2011;5:47 10.3389/fnbeh.2011.00047 21847377 PMC3148764

[35] Wang DC, Lin YY, Chen TJ, Lin HT. Motor skill learning enhances the expression of activity-regulated cytoskeleton-associated protein in the rat cerebellum. J Comp Physiol A Neuroethol Sens Neural Behav Physiol. 2014;200(11):959–66. 10.1007/s00359-014-0942-y . 25249385

[36] Ogura T, Ogata M, Akita H, Jitsuki S, Akiba L, Noda K, et al Impaired acquisition of skilled behavior in rotarod task by moderate depletion of striatal dopamine in a pre-symptomatic stage model of Parkinson's disease. Neuroscience research. 2005;51(3):299–308. 10.1016/j.neures.2004.12.006 . 15710494

[37] Akita H, Ogata M, Jitsuki S, Ogura T, Oh-Nishi A, Hoka S, et al Nigral injection of antisense oligonucleotides to synaptotagmin I using HVJ-liposome vectors causes disruption of dopamine release in the striatum and impaired skill learning. Brain research. 2006;1095(1):178–89. 10.1016/j.brainres.2006.04.039 . 16729982

[38] Hosp JA, Luft AR. Dopaminergic Meso-Cortical Projections to M1: Role in Motor Learning and Motor Cortex Plasticity. Frontiers in neurology. 2013;4:145 10.3389/fneur.2013.00145 24109472 PMC3791680

[39] Kawashima S, Ueki Y, Kato T, Matsukawa N, Mima T, Hallett M, et al Changes in striatal dopamine release associated with human motor-skill acquisition. PloS one. 2012;7(2):e31728 10.1371/journal.pone.0031728 22355391 PMC3280327

[40] Pearson-Fuhrhop KM, Minton B, Acevedo D, Shahbaba B, Cramer SC. Genetic variation in the human brain dopamine system influences motor learning and its modulation by L-Dopa. PloS one. 2013;8(4):e61197 10.1371/journal.pone.0061197 23613810 PMC3629211

[41] Soiza-Reilly M, Fossati M, Ibarra GR, Azcurra JM. Different dopamine D1 and D2 receptors expression after motor activity in the striatal critical period. Brain research. 2004;1004(1–2):217–21. 10.1016/j.brainres.2004.01.050 . 15033440

[42] McNab F, Varrone A, Farde L, Jucaite A, Bystritsky P, Forssberg H, et al Changes in cortical dopamine D1 receptor binding associated with cognitive training. Science. 2009;323(5915):800–2. 10.1126/science.1166102 . 19197069

[43] Greengard P. The neurobiology of dopamine signaling. Bioscience reports. 2001;21(3):247–69. . 11892993 10.1023/a:1013205230142

[44] Calabresi P, Gubellini P, Centonze D, Picconi B, Bernardi G, Chergui K, et al Dopamine and cAMP-regulated phosphoprotein 32 kDa controls both striatal long-term depression and long-term potentiation, opposing forms of synaptic plasticity. The Journal of neuroscience: the official journal of the Society for Neuroscience. 2000;20(22):8443–51. . 11069952 10.1523/JNEUROSCI.20-22-08443.2000 PMC6773171

[45] Nishi A, Snyder GL, Greengard P. Bidirectional regulation of DARPP-32 phosphorylation by dopamine. The Journal of neuroscience: the official journal of the Society for Neuroscience. 1997;17(21):8147–55. . 9334390 10.1523/JNEUROSCI.17-21-08147.1997 PMC6573760

[46] Dang MT, Yokoi F, Yin HH, Lovinger DM, Wang Y, Li Y. Disrupted motor learning and long-term synaptic plasticity in mice lacking NMDAR1 in the striatum. Proceedings of the National Academy of Sciences of the United States of America. 2006;103(41):15254–9. 10.1073/pnas.0601758103 17015831 PMC1622809

[47] Nairn AC, Svenningsson P, Nishi A, Fisone G, Girault JA, Greengard P. The role of DARPP-32 in the actions of drugs of abuse. Neuropharmacology. 2004;47 Suppl 1:14–23. 10.1016/j.neuropharm.2004.05.010 . 15464122

[48] Benito E, Barco A. CREB's control of intrinsic and synaptic plasticity: implications for CREB-dependent memory models. Trends in neurosciences. 2010;33(5):230–40. 10.1016/j.tins.2010.02.001 . 20223527

[49] Pittenger C, Fasano S, Mazzocchi-Jones D, Dunnett SB, Kandel ER, Brambilla R. Impaired bidirectional synaptic plasticity and procedural memory formation in striatum-specific cAMP response element-binding protein-deficient mice. The Journal of neuroscience: the official journal of the Society for Neuroscience. 2006;26(10):2808–13. 10.1523/JNEUROSCI.5406-05.2006 . 16525060 PMC6675171

[50] Oliveira AM, Abel T, Brindle PK, Wood MA. Differential role for CBP and p300 CREB-binding domain in motor skill learning. Behavioral neuroscience. 2006;120(3):724–9. 10.1037/0735-7044.120.3.724 . 16768624

[51] Kida S, Serita T. Functional roles of CREB as a positive regulator in the formation and enhancement of memory. Brain Res Bull. 2014;105:17–24. 10.1016/j.brainresbull.2014.04.011 . 24811207

[52] Bibb JA, Snyder GL, Nishi A, Yan Z, Meijer L, Fienberg AA, et al Phosphorylation of DARPP-32 by Cdk5 modulates dopamine signalling in neurons. Nature. 1999;402(6762):669–71. 10.1038/45251 . 10604473

[53] Nishi A, Bibb JA, Snyder GL, Higashi H, Nairn AC, Greengard P. Amplification of dopaminergic signaling by a positive feedback loop. Proceedings of the National Academy of Sciences of the United States of America. 2000;97(23):12840–5. 10.1073/pnas.220410397 11050161 PMC18851

[54] Ariano MA, Wang J, Noblett KL, Larson ER, Sibley DR. Cellular distribution of the rat D1B receptor in central nervous system using anti-receptor antisera. Brain research. 1997;746(1–2):141–50. . 9037493 10.1016/s0006-8993(96)01219-x

[55] Ciliax BJ, Nash N, Heilman C, Sunahara R, Hartney A, Tiberi M, et al Dopamine D(5) receptor immunolocalization in rat and monkey brain. Synapse. 2000;37(2):125–45. 10.1002/1098-2396(200008)37:2<125::AID-SYN7>3.0.CO;2-7 . 10881034

[56] Sahu A, Tyeryar KR, Vongtau HO, Sibley DR, Undieh AS. D5 dopamine receptors are required for dopaminergic activation of phospholipase C. Mol Pharmacol. 2009;75(3):447–53. 10.1124/mol.108.053017 19047479 PMC2684903

[57] Undieh AS. Pharmacology of signaling induced by dopamine D(1)-like receptor activation. Pharmacol Ther. 2010;128(1):37–60. 10.1016/j.pharmthera.2010.05.003 20547182 PMC2939266

