# Supplementary material for: Motor Skill Learning Is Associated with Phase-Dependent Modifications in the Striatal cAMP/PKA/DARPP-32 Signaling Pathway in Rodents
Source: PLoS One. 2015 Oct 21;10(10):e0140974. doi: 10.1371/journal.pone.0140974 (PMC4619563; doi:10.1371/journal.pone.0140974)
Supplement: S1 Table — (PDF) [file pone.0140974.s001.pdf]

S1 Table. Expression of Drd1 and DARPP-32 mRNAs in various cortical regions after 3 or 12 days of motor skill learning

		Drd1				DARPP-32			
		3 days		12 days		3 days		12 days	
Region	Group	Ipsi	Contra	Ipsi	Contra	Ipsi	Contra	Ipsi	Contra
mPFC	Control	174 (2.05)	163 (14.03)	149 (10.76)	146 (8.90)	191 (31.19)	189 (36.48)	150 (14.35)	189 (16.41)
	Trained	181 (14.03)	197 (15.63)	128 (9.56)	135 (5.46)	255 (13.67)	254 (14.55)	193 (5.07)	195 (7.60)
OFC	Control	176 (15.35)	176 (16.30)	157 (13.37)	155 (6.35)	209 (26.55)	206 (30.94)	206 (15.16)	209 (15.37)
	Trained	173 (13.05)	171 (12.93)	142 (10.97)	133 (14.38)	259 (15.94)	254 (21.59)	219 (2.77)	217 (6.31)
M1	Control	85 (5.74)	81 (6.29)	70 (4.85)	68 (3.34)	396 (54.08)	386 (58.00)	390 (21.63)	357 (21.23)
	Trained	81 (5.03)	78 (4.39)	60 (2.40)	58 (2.99)	505 (14.42)	514 (28.93)	389 (19.09)	382 (8.48)

All values represent means ± SEM; *n* = 5 per group. Abbreviations are as follows: ipsilateral (Ipsi), contralateral (Contra), medial prefrontal cortex (mPFC), orbitofrontal cortex (OFC), and primary motor cortex (M1).
