# Supplementary material for: Motor Skill Learning Is Associated with Phase-Dependent Modifications in the Striatal cAMP/PKA/DARPP-32 Signaling Pathway in Rodents
Source: PLoS One. 2015 Oct 21;10(10):e0140974. doi: 10.1371/journal.pone.0140974 (PMC4619563; doi:10.1371/journal.pone.0140974)
Supplement: S2 Table — (PDF) [file pone.0140974.s002.pdf]

S2 Table. Expression of Drd2 mRNA in various brain regions after 3 or 12 days of motor skill learning

Region	Group	3 days Ipsi	3 days Contra	12 days Ipsi	12 days Contra
mPFC	Control	16.9 (0.57)	17.0 (1.20)	18.4 (1.27)	17.0 (0.77)
	Trained	16.3 (0.95)	15.8 (1.03)	15.8 (1.05)	16.0 (1.57)
OFC	Control	26.1 (3.00)	26.4 (2.84)	24.3 (1.50)	26.4 (1.93)
	Trained	27.1 (3.50)	24.3 (2.78)	23.0 (1.53)	22.3 (2.27)
M1	Control	7.3 (0.56)	7.0 (0.45)	6.1 (0.41)	5.9 (0.20)
	Trained	8.2 (0.41)	7.3 (0.28)	6.0 (0.23)	6.0 (0.19)
DMS	Control	654 (21.33)	639 (22.71)	593 (18.87)	637 (22.02)
	Trained	625 (13.83)	662 (6.61)	573 (28.75)	580 (23.97)
DLS	Control	856 (23.68)	844 (23.25)	818 (24.03)	830 (17.31)
	Trained	872 (15.22)	867 (15.99)	761 (20.75)	748 (21.06)
VLS	Control	849 (28.24)	880 (23.10)	857 (13.97)	869 (26.28)
	Trained	905 (8.53)	880 (35.49)	810 (27.86)	774 (20.78)
AcbC	Control	369 (21.07)	376 (24.19)	305 (19.89)	284 (10.59)
	Trained	346 (27.43)	344 (23.95)	325 (24.89)	302 (25.21)
AcbSh	Control	398 (28.62)	394 (38.76)	308 (28.83)	311 (17.24)
	Trained	365 (43.50)	358 (23.59)	329 (28.45)	299 (37.44)

All values represent means ± SEM; n = 5 per group. Abbreviations are as follows: ipsilateral (Ipsi), contralateral (Contra), medial prefrontal cortex (mPFC), orbitofrontal cortex (OFC), primary motor cortex (M1), dorsal lateral striatum (DLS), dorsal medial striatum (DMS), ventrolateral striatum (VLS), nucleus accumbens core (AcbC), and nucleus accumbens shell (AcbSh).
