# Supplementary material for: Motor Skill Learning Is Associated with Phase-Dependent Modifications in the Striatal cAMP/PKA/DARPP-32 Signaling Pathway in Rodents
Source: PLoS One. 2015 Oct 21;10(10):e0140974. doi: 10.1371/journal.pone.0140974 (PMC4619563; doi:10.1371/journal.pone.0140974)
Supplement: S3 Table — (PDF) [file pone.0140974.s003.pdf]

S3 Table. Levels of total and phosphorylated DARPP-32 and CREB in striatum after 12 days of motor skill learning

Group	p-Thr34-DARPP-32	p-Thr75-DARPP-32	p-Ser97-DARPP-32	Total DARPP32	p-Ser133-CREB	Total CREB
Control	100.00 (8.76)	100.00 (7.91)	100.00 (5.85)	100.00 (2.06)	100.00 (6.50)	100.00 (2.62)
Trained	100.94 (12.67)	87.10 (7.14)	101.98 (5.24)	105.84 (2.28)	122.85 (8.35)	94.75 (3.98)

All values represent means ± SEM; *n* = 6-10 per group.
